# Equity in Health-Seeking Behavior of Groups Using Different Transportations

**DOI:** 10.3390/ijerph19052765

**Published:** 2022-02-27

**Authors:** Fangye Du, Jiaoe Wang, Yu Liu, Zihao Zhou, Haitao Jin

**Affiliations:** 1 Key Laboratory of Regional Sustainable Development Modeling, Institute of Geographic Sciences and Natural Resources Research, Chinese Academy of Sciences, Beijing 100101, China; dufy.18b@igsnrr.ac.cn; 2 College of Resources and Environment, University of Chinese Academy of Sciences, Beijing 100049, China; 3 Institute of Remote Sensing and Geographical Information Systems, School of Earth and Space Sciences, Peking University, Beijing 100871, China; liuyu@urban.pku.edu.cn; 4 Concord College, Shrewsbury SY5 7PF, UK; stevenzhouzihao@outlook.com; 5 School of Computer, Beijing Information Science and Technology University, Beijing 100101, China; jinht@bistu.edu.cn

**Keywords:** healthcare accessibility, equity, comparative analysis, smart card data, taxi trajectory data

## Abstract

The equity of health-seeking behaviors of groups using different transportations is an important metric for health outcome disparities among them. Recently, smart card data and taxi trajectory data have been used extensively but separately to quantify the spatiotemporal patterns of health-seeking behavior and healthcare accessibility. However, the differences in health-seeking behavior among groups by different transportations have hitherto received scant attention from scholars. To fill the gap, this paper aimed to investigate the equity in health-seeking behavior of groups using different transportations. With sets of spatial and temporal constraints, we first extracted health-seeking behaviors by bus and taxi from smart card data and taxi trajectory data from Beijing during 13–17 April 2015. Then, health-seeking behaviors of groups by bus and taxi were compared regarding the coverage of hospital service areas, time efficiency to seek healthcare, and transportation access. The results indicated that there are inequities in groups using different travel modes to seek healthcare regarding the coverage of hospital service areas, time efficiency to seek healthcare, and transportation access. They provide some suggestions for mode-specific interventions to narrow health disparity, which might be more efficient than a one-size-fits-all intervention.

## 1. Introduction

Equity of healthcare accessibility is essential for narrowing the disparity of health outcomes [1,2,3]. Patients’ travel mode choice is closely related to their socio-economic characteristics. For instance, low-income patients and the elderly are prone to choose public transits when seeking healthcare, whereas high-income patients prefer private cars or taxis [4,5,6]. Understanding the health-seeking behaviors of groups using different transportations to seek healthcare is key to effective interventions to precisely evaluate accessibility and narrow health outcomes among them.

The past decades have witnessed an exponential growth of scientific research that investigates human activities and urban dynamics. Traditionally, travel survey data was widely used to explore travel behavior, human daily activities, activity space, etc. [7,8]. Such data have long been criticized for their limited sample size, high cost, and inefficiency [9,10]. The recent advances in data collection and analysis methods allow for investigation into human mobilities and urban dynamics with a higher resolution using big data, such as smart card, taxi trajectory, and mobile phone data [11,12,13]. Different datasets have different data structures and reflect different dimensions of human mobility; thus, divergences in human mobility patterns can be highlighted from different datasets. Nevertheless, much of the current literature analyzes only a single type of dataset [14,15,16]. It is not until recently that some studies started to explore the divergence [17,18,19,20].

Taxi and bus are two popular travel modes to access healthcare in metropolitan areas. A recent survey on health-seeking behavior in Beijing, China, reported that about 40% of patients went to hospitals by bus and taxi [21]. With the application of big data on investigating travel behaviors, methods of inferring health-seeking behavior from smart card data and taxi trajectory data have been proposed and validated. Moreover, health-seeking trips from smart card data and taxi trajectory data have been widely used to investigate hospital service areas, healthcare accessibility, etc. [10,22,23]. Despite fruitful research outcomes, the differences among health-seeking behavior among groups using different transportations have hitherto received scant attention from scholars. As indicated by [18,19], differences in travel behavior using varied travel modes exist. These differences emphasize the need for knowledge regarding how the travel behaviors vary among groups using different travel modes, based on which mode-specific interventions, rather than a one-size-fits-all intervention, should be designed to improve overall access to healthcare. Without a consideration of health-seeking behavior of groups using different transportations, health planners may misidentify populations with low access to healthcare and consequently devise less-effective interventions. Therefore, this paper contributes to the existing literature by investigating the equity in health-seeking behaviors of groups by different transportations. Using the effective methods widely used in previous studies, we first extracted health-seeking behaviors from smart card data and taxi trajectory data in Beijing, respectively. Then, the comparative analysis was conducted in three aspects: coverage of the hospital service area, time efficiency to seek healthcare, and transportation access. The results provide some suggestions for improving healthcare accessibility and narrowing health disparity among different groups.

## 2. Literature Review

In this section, we first reviewed the literature on travel behavior investigated through different transport records. Then, related works exploring the health-seeking behavior of patients using different travel modes were summarized.

### 2.1. Travel Behavior and Transportation

Each transportation mode has its own unique characteristics regarding service deployment, cost, and customers [24,25,26]. Public transit, e.g., bus and metro, provide only station-to-station services. To reach the station and to reach the destination from stations, other travel modes (such as walking and bicycling) are required, which might have implications for the person’s physical strength [21,27]. Public transit is often preferred by vulnerable groups, such as low-income groups and older people. Unlike public transit, taxis provide a door-to-door service, which is more comfortable and flexible, although at a higher cost [10,22,28]. Private cars are not only comfortable but also flexible with time. However, limited parking spaces and traffic jams restrict private car use in metropolitan cities [29].

People’s travel mode choice is the outcome of the interactions among various factors, such as demographics, socio-economic status, trip characteristics, trip purposes, and service deployment [25,30]. For example, Böcker et al. (2017) indicated that the travel mode choice and associated factors are varied across different age cohorts [26]. Kim and Ulfarsson (2004) found that older people’s travel mode choice is sensitive to trip purposes. In terms of trip purposes, transits are less frequently used by those doing errands and shopping [31]. Du et al. (2021) reported that adults are more likely to use cars when making a long trip to seek healthcare [6]. Regarding service deployment, Commins and Nolan (2011) found that higher public transport availability and limited parking facilities promote public transit use for work trips [24].

With the rapid developments of sensing and data collecting technologies, big data, such as smart card data, taxi trajectory data, and mobile phone data, have been widely used to support studies of human mobility, travel patterns, and urban dynamics [12,32,33]. However, they often represent the travel patterns of different groups and reflect different dimensions of human activity [17,18]. Smart card data records the travel trajectories of passengers of bus or subway systems [21,27]. Unique card number for passengers enable researchers to track the trip chains at the individual level. It has been widely used to explore the change in travel patterns over time, trip purposes, commuting patterns, and the jobs–housing balance [11,27,34]. Different from smart card data, taxi trajectory data records the movements of taxis and their occupancy status over space and time. It cannot track the mobility of specific passengers [10]. Such data has been widely used to present a detailed analysis of urban traffic conditions, spatial interactions, and the distribution of pick-up and drop-off points [35,36]. Mobile phone data mainly captures the activity hotspots and mobility patterns for large numbers of phone users at the base station scale without the trajectories of a single individual [37,38,39]. Despite this, the majority of previous studies only quantify human mobility and travel patterns with a single type of data [14,15,16]. Realizing that different datasets often reflect different dimensions of human activity, a few studies have begun to explore the differences among these datasets in investigating human mobility and urban dynamics [17,40]. For instance, Zhang et al. (2018) proposed an analytical framework to compare urban mobility patterns extracted from smart card data and taxi trajectory data [19]. It has been demonstrated, with Singapore as a case study, that different datasets reveal different urban mobility patterns.

### 2.2. Health-Seeking Behavior and Transportation

Traditionally, patient registry data are regarded as an ideal dataset to explore characteristics of people’s health-seeking behavior [41]. Such data, however, can be extremely difficult to obtain due to its confidentiality and sensitivity. The household travel survey data, collected by government agencies or research institutes, are an alternative to the patient registry data. With survey data, factors associated with patients’ travel mode choices are widely explored [4,5]. Du et al. (2021) explored the determinants of travel mode choice for healthcare-seeking of non-elderly and elderly patients in Beijing, China [6]. Related studies all indicated that patients with different socio-economic characteristics are prone to choose different travel modes [26,42]. Low-income patients and the elderly are prone to choose public transits when seek healthcare, whereas high-income patients prefer private cars or taxis [43]. Therefore, understanding the travel behaviors with different transportations is not only significant for transport planning but also helps to narrow the disparity of healthcare accessibility among different groups. However, travel patterns, especially spatial patterns, are rarely investigated with survey data due to a limited sample size and poor statistical representativeness [23].

Recently, smart card data and taxi trajectory data have been validated to be effective datasets to investigate health-seeking behavior [10,44]. For example, Du et al. (2020) proposed a method to extract transit-based health-seeking trips from smart card data, taking Beijing as a case study for demonstration and validation [21]. Pan et al. (2018) identified health-seeking trips from taxi trajectory data and proposed an integrated catchment area with actual taxi trips in Shenzhen [23]. With health-seeking behavior extracted from taxi trajectory data, Kong et al. (2017) characterized hospital service areas and patients’ distribution [22]. However, they are often used separately to explore the spatio-temporal patterns of health-seeking behavior. To date, little attention has been paid to the divergence among health-seeking behaviors with different transportations.

## 3. Research Design

### 3.1. Data Collection and Processing

**Figure 1:**
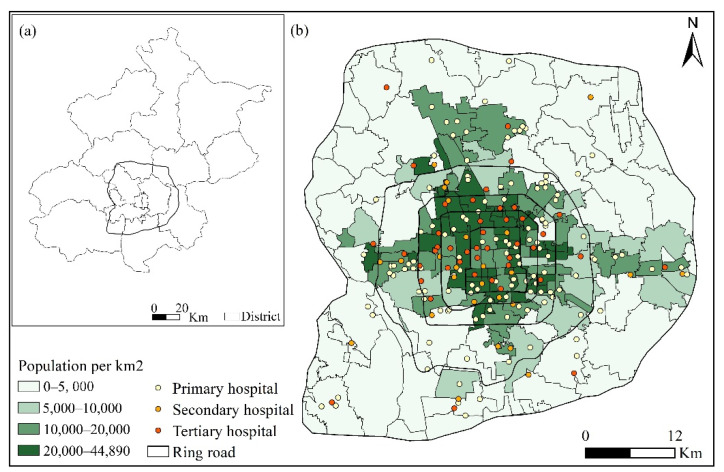
(**a**) Study area within the sixth ring road of Beijing; (**b**) distribution of primary, secondary, and tertiary general hospitals and the population density in subdistricts.

Beijing, the capital of China, has an extensive public transportation network, especially in the core urban areas. The urban structure of Beijing is distinctly monocentric (including the second, third, fourth, fifth, and sixth ring roads). The areas within the sixth ring road are recognized as the core urban area [10], and this was therefore our study area. Meanwhile, the subdistrict was selected as the statistical unit, and there are a total of 171 subdistricts within the sixth ring road of Beijing (Figure 1). To demonstrate the differences between health-seeking behaviors of groups by bus and taxi, datasets regarding hospital facilities, smart card data, and taxi trajectory data were collected, described as follows.

#### 3.1.1. Hospital Data

**Figure 2:**
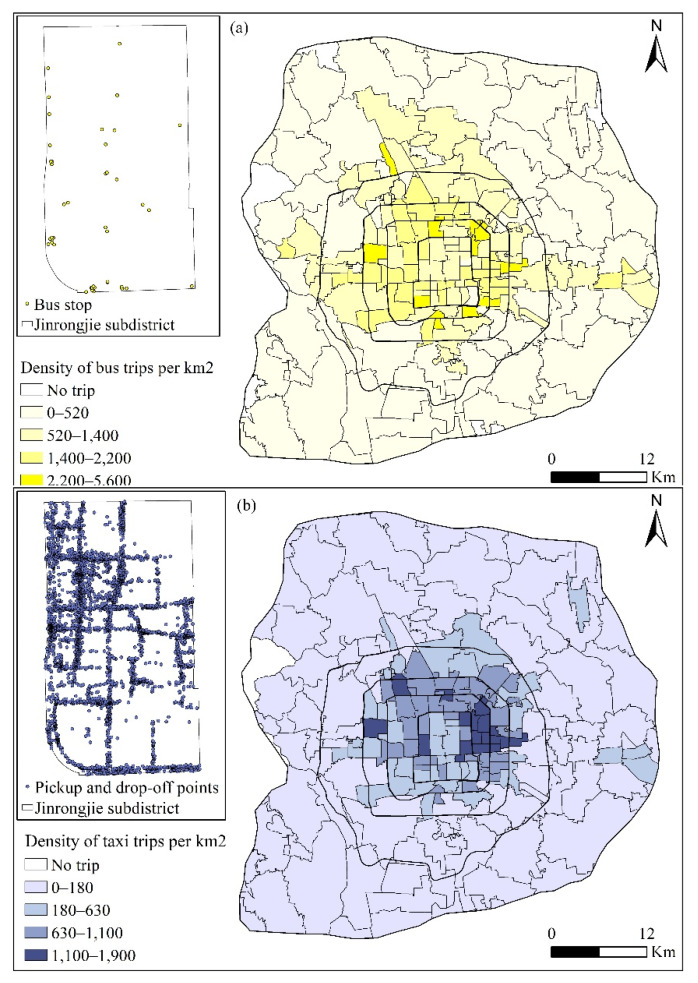
(**a**) Distribution of bus trips by subdistrict and (**b**) distribution of taxi trips.

According to their functions, hospitals can be classified into general and specialized hospitals. General hospitals provide various medical services, whereas specialized hospitals only serve patients with certain types of diseases, e.g., cardiovascular disease, or specific groups of patients, e.g., children and pregnant woman. Specialized hospitals were excluded from this analysis because they do not serve the general population. General hospitals are further classified into three tiers: primary, secondary, and tertiary hospitals. Tertiary hospitals provide the most notable specialists, professional skills, and advanced equipment. We obtained from Baidu Maps a total of 227 general hospitals within the sixth ring road of Beijing, including 147 primary, 30 secondary, and 50 tertiary hospitals. (Figure 2). Since most departments in hospitals are out of service during the weekend, we collected smart card data and taxi trajectory data for five consecutive working days in a week (both from 13–17 April 2015).

#### 3.1.2. Public Transport Records

All bus lines in Beijing have adopted a distance-based fare system since January 2015. The system records trip information when passengers swipe their card upon getting on and off the bus. Information recorded includes card ID, operation date, get-on stop (latitude and longitude), get-on time, get-off stop (latitude and longitude), and get-off time. Examples of smart card records are shown in Table 1. From 13–17 April 2015, 39.5 million records of smart card data were collected from 7.8 million card users. Figure 2a describes the density of bus trips by subdistrict, which was shown to be highly related to the distribution of bus stops. To zoom in on the distribution of bus stops, the *Jinrongjie* subdistrict, located in the core urban areas, was selected as an example (Figure 2a).

#### 3.1.3. Taxi Trajectory Data

A taxi trajectory system collects information on each taxi’s location (latitude and longitude), time, status (vacant or occupied), and speed every 10 s (Table 2). Based on this information, the pick-up and drop-off information for each trip can be identified. We thus obtained the vectors from (x_O_, y_O_, t_O_) to (x_D_, y_D_, t_D_), where x and y represent the latitude and longitude of points, and t was the time of drop off or pick up. Consistent with smart card data, taxi trajectory data from 13–17 April 2015 were collected. There were about 8 million taxi trips generated by 19,700 taxis. Figure 2b depicts the density of taxi trips by subdistricts, and we found that the taxi trips were mainly concentrated in the areas within the fourth ring road. As observed from the Jinrongjie subdistrict, the pick-up and drop-off points were denser for taxis than buses, since taxis can stop almost anywhere along the road.

### 3.2. Methods

#### 3.2.1. Inferring Health-Seeking Trips from Smart Card Data

As bus stops serve all facilities nearby, getting off at a bus stop near a hospital does not necessarily mean that the passenger is heading for the hospital. It was thus necessary to further confirm health-seeking behavior from other sources of information. Each activity has its unique spatial, temporal, and behavioral characteristics; thus, a set of constraints proposed by Du et al. (2020) was used to extract health-seeking trips from smart card data [21]. The data processing was done on the Python platform. The specific method is described as follows.

The trip chain was first constructed for each passenger, including several trip legs based on their unique card ID. All transfer activities, identified by an activity duration shorter than 20 min and displacement shorter than 500 m, were removed to identify the origins and destinations of trips. Then, trip chains containing a pair of get-on and get-off stops that were both within walking distance (500 m) of the same health facility were extracted. Furthermore, only trips with a duration between 50 and 300 min were included. In addition, the maximum frequency of health-seeking was set as once every three days, which is consistent with the health-seeking frequency of patients with chronic diseases. As the trip purpose of companions is not to seek healthcare, only one trip was utilized for multiple instances of the same health-seeking trip (regarding boarding and alighting time at all stops, origins, destinations and the time of stay in healthcare facilities). With a set of spatial, temporal, and behavioral constraints, about 62,000 health-seeking trips were identified from 5.4 million trip chains.

#### 3.2.2. Extracting Health-Seeking Trips from Taxi Trajectory Data

**Figure 3:**
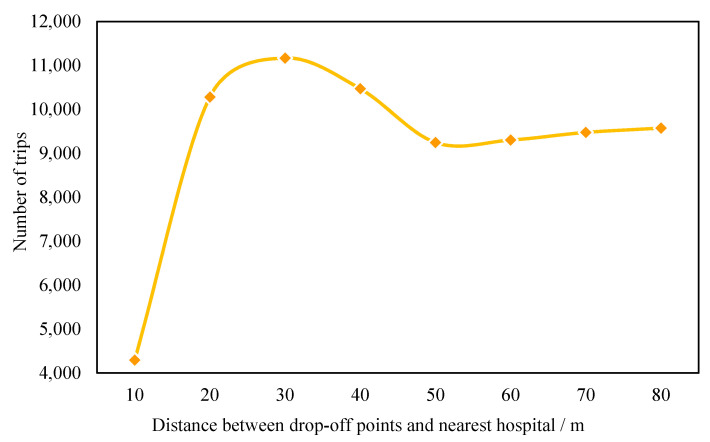
Frequency distribution of distance between drop-off points and nearest hospitals.

The locations of drop-off and pick-up points can reflect facilities where passengers visit. The distance between the locations of drop-off/pick-up points and facilities is a good metric for the purpose of the trip. In this case, the distance between a drop-off point and the nearest hospital was set to extract trips to hospitals (also named health-seeking trips by taxi) from taxi trajectory data. The frequency distribution of the distance between drop-off points and the nearest hospital (hospital gate) is shown in Figure 3. The results indicate that the number of trips increased in the range of 0 to 40 m and then decreased in the range of 40 to 50 m. When the distance was longer than 50 m, the number of trips began to increase, as trips to other destination facilities might have been included. Therefore, trips with drop-off points within 0 and 50 m from hospital gates were identified as health-seeking trips. It is worth noting that trips with pick-up points within the range were not included in this study to avoid double counting. For comparison purposes, the number of health-seeking trips were multiplied by five. Therefore, both smart card data and taxi trajectory data represent total samples of trips. Based on this method, there were about 130,000 health-seeking trips by taxi between 13–17 April 2015. The data processing was also done on the Python platform.

**Figure 4:**
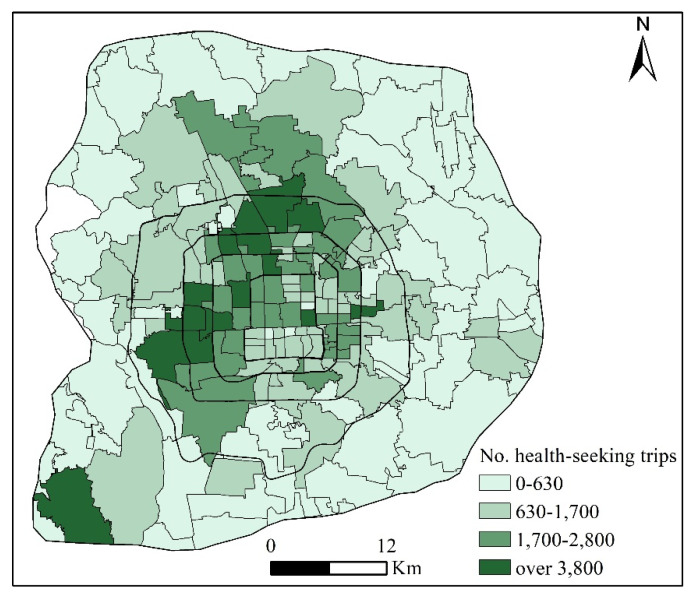
Distribution of the origins of health-seeking trips by bus and taxi.

In total, about 192,000 health-seeking trips were extracted from smart card data and taxi trajectory data in Beijing between 13–17 April 2015. Figure 4 depicts the distribution of health-seeking trips, which were highly consistent with the distribution of hospitals. Specifically, health-seeking trips tended to be concentrated in the core areas of the whole city and the core areas of subdistricts in the periphery, such as the Liangxiang subdistrict of the Fangshan district located in the southwestern corner of the city near the sixth ring road.

### 3.3. Research Framework

**Figure 5:**
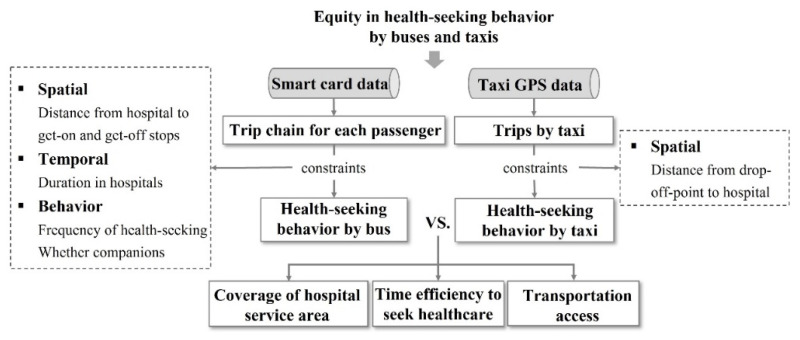
Analytical framework. Source: designed by the author.

As indicated in existing studies, individuals’ travel mode choices are often based on their demographic characteristics, economic statuses, physical activeness, and built environment nearby [6,45]. For instance, individuals who use buses need to complete the journey from bus stops to hospitals and from home to bus stops by other travel modes, such as walking and bicycling. It requires more physical strength but is more economically affordable. Compared to buses, taxis are more flexible regarding location and time but have higher fees. Patients with physical limitations and high incomes are more likely to choose taxis. For this reason, there might be inequity in the health-seeking behaviors of groups by bus and taxi. In previous attempts on exploring health-seeking behavior and healthcare accessibility, however, the same healthcare demand and hospital service areas were applied for different transportations [45]. In this circumstance, populations for which healthcare is inaccessible might be misidentified. Therefore, this study aims to explore the equity in health-seeking behaviors of groups using different travel modes. Specifically, health-seeking trips were first extracted from the smart card and taxi trajectory data with different constraints due to the different data structures. The coverage of the hospital service area, time efficiency to seek healthcare, and transportation access are three aspects that are closely related to healthcare accessibility. Thus, health-seeking behaviors of groups by bus and taxi were compared in the three aspects mentioned above. The analytical framework is shown in Figure 5.

## 4. Results and Discussion

As patients have varied demographic characteristics, economic statuses, and physical activeness, they might be inequity in health-seeking behaviors, such as time efficiency to seek healthcare and transportation access. This study illustrates the equity of health-seeking behavior by bus and taxi in three aspects: coverage of hospital service area, time efficiency to seek healthcare, and transportation access.

### 4.1. Coverage of Hospital Service Area

Depending on the transportation mode, hospitals often provide services for the population in only some subdistricts. For example, patients located in the subdistricts without bus services, or with inconvenient access to them, are less likely to seek healthcare by bus. Therefore, hospital service areas estimated by smart card data do not cover these subdistricts. As demarcating hospital service areas is an important part of assessing hospital accessibility, the coverages of hospital service areas estimated by smart card data and taxi trajectory data were compared to preliminarily understand their differences. The top 10 hospitals regarding the coverage of hospital service areas are listed in Table 3. For both smart card data and taxi trajectory data, the top 10 hospitals regarding coverage were all tertiary hospitals. It is reasonable because advanced technology and professional doctors attract a great number of patients [45]. Furthermore, we found that hospital service areas weere varied according to the transportation mode, with the coverage of service area by taxis being greater than that by buses, consistent with the wider spatial coverage of taxi services.

**Figure 6:**
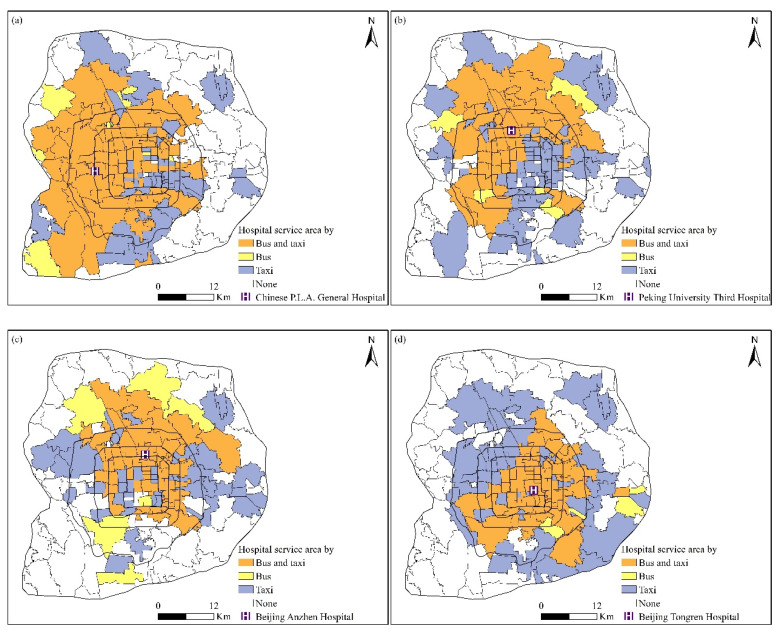
Comparison of the hospital service areas estimated by smart card data and taxi trajectory data for (**a**) Chinese P.L.A. General Hospital, (**b**) Peking University Third Hospital, (**c**) Beijing Anzhen Hospital, and (**d**) Beijing Tongren Hospital.

To further understand the differences in hospital service areas by bus and taxi, the service areas of four representative hospitals in Beijing estimated by smart card data and taxi trajectory data were compared. Chinese P.L.A. General Hospital, Peking University Third Hospital, Beijing Anzhen Hospital, and Beijing Tongren Hospital were selected as the representative hospitals, as their service area coverages are large, making them suitable for estimation from both smart card data and taxi trajectory data. As shown in Figure 6, the hospital service areas for the two datasets are both centered around the corresponding hospitals. More importantly, the hospital service area by taxi has a wider coverage than that by bus. An explanation might be that the pick-up and drop-off points of taxis are more flexible compared to buses, as they can serve subdistricts with no bus services. In contrast to other activities, those who seek healthcare often have physical limitations, which promote taxi use. Spatially, hospital service areas by bus and taxi overlap in subdistricts immediately surrounding the hospitals. In addition, hospital service areas by taxi also cover the subdistricts surrounding the overlapped subdistricts. Meanwhile, hospital service areas by bus cover subdistricts that have convenient bus services but are relatively far away from hospitals.

### 4.2. Time Efficiency to Seek Healthcare

The travel time to hospitals was used here to measure patients’ time efficiency to seek healthcare. Taxis provide door-to-door services, and passengers’ drop-off and pick-up points are often near their origins and destinations. The difference between the drop-off time and pick-up time is roughly equal to the travel time. Buses, however, only provide station-to-station services. For bus trips, door-to-door travel time must also include the walking time toward and away from bus stops, waiting time for a bus, and transfer time between different bus routes. The acceptable walking distance of bus passengers in Beijing is 500 m, and the walking speed is about 5.5 km/h. In light of this information, passengers’ walking time toward and away from bus stops were assigned randomly with a range of 1–5 min. As buses in Beijing have a 15–20 min headway, we assigned a random value between 1 and 20 min to each passenger’s waiting and transferring between different bus routes. The travel distance of each health-seeking trip by bus was calculated as the sum of the network distance between the on-station and off-station, the distance between the origin and on-station, and the distance between the off-station and hospital. As the acceptable walking distance of bus passengers in Beijing is 500 m, the distance between the origin and on-station and the distance between the off-station and hospital were assigned randomly in the range of 0–500 m. Trips by taxi, on the other hand, were calculated as the network distance between the origin and destination.

**Figure 7:**
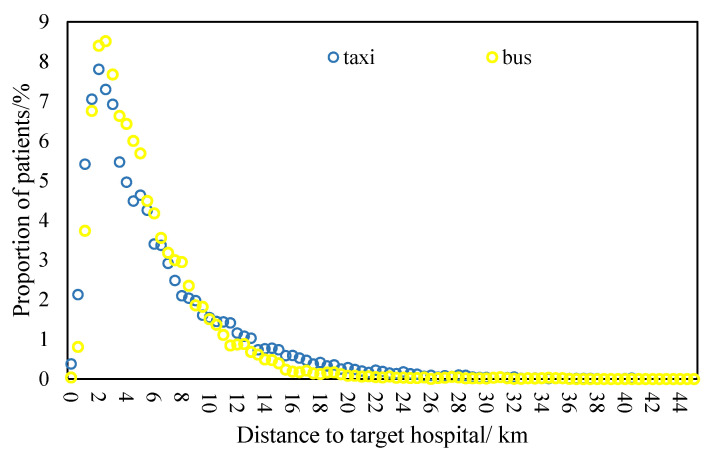
Distance decay of health-seeking trips by bus and taxi.

Statistically, the average travel time of health-seeking behavior by bus was 24.6 min, which was 3.4 min longer than that by taxi. However, the average travel distance of health-seeking trips by bus was about 5.6 km, which was 0.9 km shorter than by taxi. This indicates that taxis are more efficient for health-seeking behavior compared to buses. Furthermore, a distance-decay effect of health-seeking behavior by both travel modes was present. For both taxis and buses, the distance decay phenomenon occurs when the travel distance is longer than a certain value, namely 2 km for taxis and 2.5 km for buses. Meanwhile, the decay coefficient of health-seeking behavior by bus is much higher than that by taxi (Figure 7). This finding is consistent with previous literature, such as the study by [19]. One explanation of this could be that the travel distance of health-seeking trips is more concentrated.

**Figure 8:**
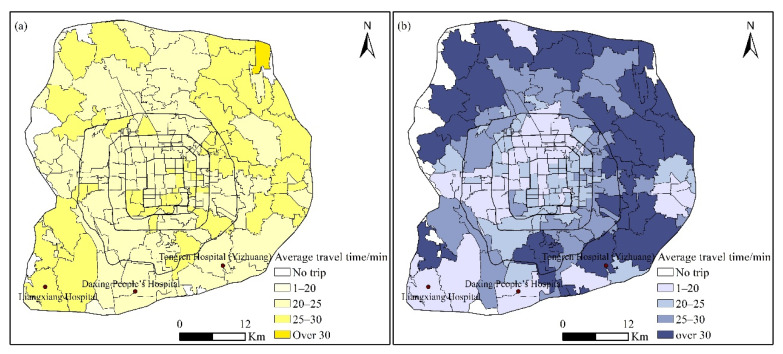
Distribution of average travel time of health-seeking behavior by bus (**a**) and taxi (**b**).

Spatially, the subdistricts located in the southwestern corner, northeastern corner, and northwestern corner between the fifth and sixth ring roads had a long travel time when seeking healthcare by bus (Figure 8a). One simple reason is that patients in these subdistricts seek healthcare in hospitals far away from them. Surprisingly, some subdistricts in the core urban area also had relatively long average travel times to hospitals. Hospital choice and traffic congestion might be two main contributors to this phenomenon. Figure 8b depicts the spatial distribution of the average travel time of health-seeking behavior by taxi, which shows a distinct monocentric structure. The subdistricts located in the core urban areas had a shorter average travel time than those in the periphery. In addition, the subdistricts in the periphery areas with tertiary hospitals, such as subdistricts near Liangxiang Hospital, Daxing People’s Hospital, and Tongren Hospital (Yizhuang), also had a short travel time, as patients in these subdistricts are more likely to seek healthcare in the hospitals nearby.

### 4.3. Transportation Access

**Figure 9:**
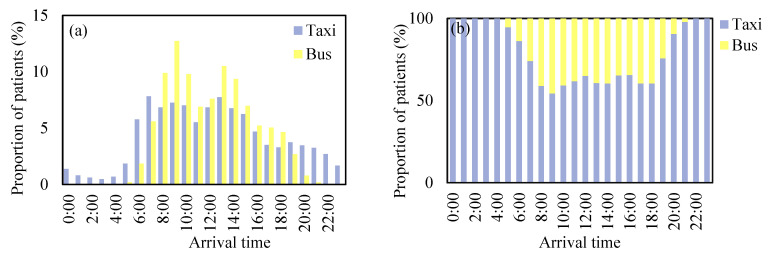
Hourly variation of (**a**) health-seeking trips extracted from smart card data and taxi trajectory data, and (**b**) travel mode preference.

Travel mode choice is an important indicator for transportation access. Here, the temporal dynamics and spatial patterns of travel mode choice were investigated. Health-seeking behaviors by bus and taxi each showed distinct temporal dynamics. As shown in Figure 9a, the arrival time of health-seeking behavior by bus showed a bimodal distribution, with peaks at 8:00–10:00 and 13:00–15:00. For health-seeking behavior by taxi, patients might arrive at hospitals at any time in a day, and there was no distinct peak hour. By comparing the proportion of patients seeking healthcare with the two travel modes by the hour, we found that taxi is the dominant travel mode for health-seeking behavior, with its proportion consistently over 50%, even in the peak hours of health-seeking by bus (Figure 9b). The reason might be that individuals who seek healthcare are often with low physical activeness and are prone to choose comfort transportation [6].

**Figure 10:**
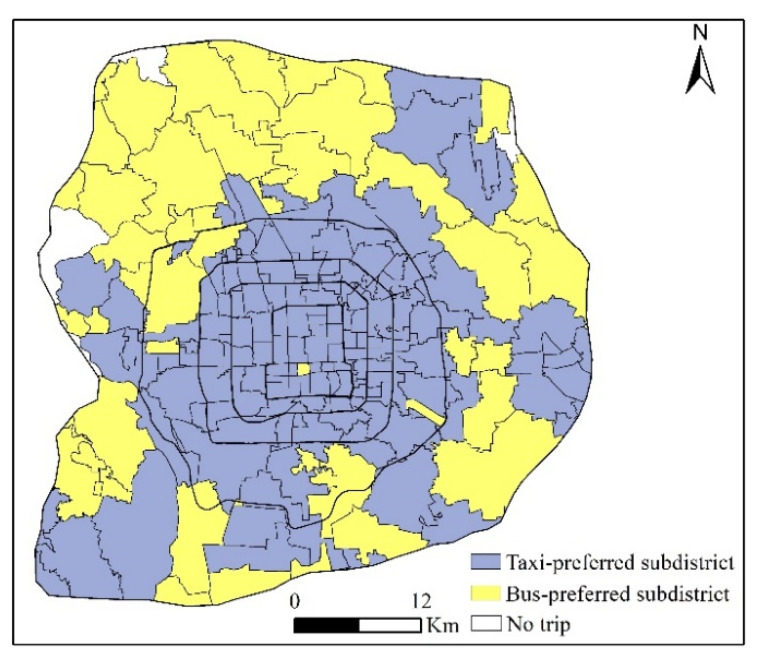
Travel mode preference by subdistrict.

For visualization purposes, the number of health-seeking trips by bus and taxi was compared in each subdistrict to detect taxi-preferred subdistricts (the number of taxi trips exceeded bus trips) and bus-preferred subdistricts (the number of bus trips exceeded taxi trips). As shown in Figure 10, taxi-preferred subdistricts were mainly concentrated in the core areas of the whole city and the core areas of the districts in the periphery, whereas bus-preferred subdistricts were in the periphery. The core areas are characterized by both convenient bus and taxi services. As patients who seek healthcare are more likely to have physical limitations, they would prefer taxi services, which are comfortable and flexible. Patients located in the periphery areas are often far away from hospitals; thus, they have a trade-off between transportation cost and comfortable experiences when choosing a travel mode. Taxis are charged by trip distance, which is about 2.3 RMB/km in Beijing. Therefore, taking a long trip would incur a high cost. As a result, patients in areas with convenient bus services, especially when there are direct bus routes to destination hospitals, might prefer buses to avoid high transportation costs. However, those located in areas with inconvenient bus services must take a taxi to seek healthcare.

### 4.4. Policy Implications

According to the results, several suggestions and policy implications could be proposed to narrow healthcare disparity. For health planners, more light should be shed on the spatial coverage of hospital service areas by different travel modes. The demarcation of hospital service areas is the basis for investigating the total demand for a certain hospital, and thus, it is crucial to assess healthcare accessibility. Depending on travel mode, the service area of hospitals is often very different. In previous practices, however, the hospital service area of only one travel mode was considered in the evaluation of healthcare accessibility, which might lead to misidentification of populations with low access to healthcare, leading to less-effective interventions. For transportation planners, more direct bus routes should be planned to connect populations in subdistricts far away from hospitals, especially in the peak hours of health-seeking. The level of convenience of transportation services is one of the determinants of travel mode choice to seek healthcare. For example, healthcare demands through bus services and hospital service areas estimated by smart card data are both closely related to the distribution of bus services, especially direct bus routes. In Beijing, top hospitals are often located in the core urban areas with heavy traffic, and thus, the long travel time caused by traffic congestion is one of the main contributors to low healthcare accessibility. Since promoting public transportation use can be regarded as one efficient way to reduce traffic congestion, this study suggests the operation of more direct bus routes between hospitals and distant subdistricts to narrow the disparities between groups that use buses and taxis to seek healthcare.

### 4.5. Limitations

Some limitations in this paper are worthy of discussion. First, with the validated method proposed by Du et al. (2020), we set spatial, temporal, and behavioral constraints to extract health-seeking behavior from smart card data [21]. We extracted health-seeking trips from taxi trajectory data with widely used methods in the research of [22,23]. It is inevitable that some activities near the hospitals were included. However, they were the minority, and the results are still effective in providing a global estimation of the spatio-temporal patterns of health-seeking behavior, rather than accurate estimation. The second issue is the representativeness of taxis and buses. Buses and taxis are two popular travel modes in the metropolis of China. A recent survey on health-seeking behavior in Beijing, China, reported that about 40% of patients went to hospitals by bus and taxi [21]. Most well-known hospitals in Beijing with a long history are located in the inner city, where the parking infrastructure and space are lacking. For example, Peking University Third Hospital has 400 parking spaces but serves more than 7000 patients per day. Furthermore, there are few public parking spaces around the hospital. In such cases, parking spaces cannot meet the demands of patients. Third, limited by data availability, we only explained the differences in health-seeking behavior among patients using different transportation with the evidence from existing studies, rather than through quantitative methods. In future work, the results could be further explained with data from a health-seeking behavior survey.

## 5. Conclusions

Characterized by the large volume and high spatial and temporal precision, big data have been widely used in investigations of human mobility patterns, urban dynamics, and more. Moreover, existing studies verified that different transportation records reflect the different dimensions of human movements. Despite this, the divergences in human mobility patterns using different transportations have been highlighted only in the very recent studies. Regarding health-seeking behavior, transportation records, such as smart card data and taxi trajectory data help us to uncover spatiotemporal patterns and quantify healthcare accessibility. The differences among health-seeking behavior with different types of transport remains poorly understood. Without these considerations, health planners may misidentify populations with low access to healthcare and devise less-effective interventions. This study contributes to the existing literature by exploring the differences in health-seeking behaviors among groups using different transportations. With health-seeking behaviors extracted from smart card data and taxi trajectory data as an example, the differences were explored with a focus on coverage of hospital service areas, time efficiency to seek healthcare, and transportation access.

We found inequities in groups using different travel modes to seek healthcare regarding the coverage of the hospital service area, time efficiency to seek healthcare, and transportation access. The specific findings were as follows. First, hospitals provide wider service areas for those taking taxis to seek healthcare as compared with those by bus. Second, health-seeking behaviors by taxis are more time-efficient than buses, as health-seeking behavior by taxi has a relatively long travel distance and short travel time as compared with the bus. Third, the transportation access varies over space. Overall, the transportation access in the core urban areas is higher than that in the peripherical areas. In the periphery areas, patients in subdistricts with convenient bus services might prefer buses, whereas those who cannot access convenient bus services are more likely to seek healthcare with taxis.

This work provides insights into the divergences in health-seeking behavior of different groups: those seeking healthcare by bus and taxi. The results offer some suggestions for narrowing the health disparity among different groups by bridging the gaps in their healthcare accessibility. Although health-seeking behaviors in Beijing were taken as a case study, the analytical framework proposed in this study could be applied in other cities.

## Figures and Tables

**Table 1:** Examples of smart card records in the bus system.

Card ID	Get-On Stop			Get-Off Stop		
	Latitude	Longitude	Time	Latitude	Longitude	Time
0002	116.489	39.968	10:44:00	116.204	39.927	11:12:00
2092	116.468	39.952	16:10:10	116.489	39.968	17:20:20
0012	116.470	39.867	8:10:00	116.450	39.856	8:30:20
0050	116.398	39.975	11:30:35	116.444	39.711	12:30:40

**Table 2:** Examples of taxi trajectory data.

Taxi ID	Pick-Up Point			Drop-Off Point		
	Latitude	Longitude	Time	Latitude	Longitude	Time
febc89a6	116.582	40.079	2 April 2015 7:35	116.352	39.980	2 April 2015 8:00
98ea748	116.249	39.984	2 April 2015 15:06	116.272	39.955	2 April 2015 15:24
de864n	116.468	39.952	2 April 2015 21:03	116.489	39.968	2 April 2015 22:06

**Table 3:** The top 10 hospitals accessed by subdistrict, extracted from smart card data and taxi trajectory data.

	Smart Card Data			Taxi Trajectory Data		
Rank	Hospital	Hospital Level	No. Subdistricts	Hospital	Hospital Level	No. Subdistricts
1	Beijing Shijitan Hospital	Tertiary	70	Beijing Xiehe Hospital (East)	Tertiary	114
2	Chinese P.L.A. General Hospital	Tertiary	54	Beijing Tongren Hospital	Tertiary	112
3	China-Japan Friendship Hospital	Tertiary	66	Peking University Third Hospital	Tertiary	110
4	Beijing Anzhen Hospital	Tertiary	64	Chinese P.L.A. General Hospital	Tertiary	110
5	Beijing Tongren Hospital	Tertiary	64	Beijing Xiehe Hospital (West)	Tertiary	108
6	Beijing Xiehe Hospital (East)	Tertiary	59	Beijing Xuanwu Hospital	Tertiary	106
7	Peking University People’s Hospital	Tertiary	59	China-Japan Friendship Hospital	Tertiary	105
8	Beijing Friendship Hospital	Tertiary	54	Beijing Chaoyang Hospital	Tertiary	103
9	Beijing Chaoyang Hospital	Tertiary	52	Peking University First Hospital	Tertiary	102
10	Peking University Third Hospital	Tertiary	51	Beijing Anzhen Hospital	Tertiary	100

## Data Availability

The data is not available.

## References

[1] Vincent D., San S.M. (2018). Is healthcare really equal for all? Assessing the horizontal and vertical equity in healthcare utilisation among older Ghanaians. Int. J. Equity Health.

[2] Panagiotopoulos G., Kaliampakos D. (2018). Accessibility and spatial inequalities in Greece. Appl. Spat. Anal. Policy.

[3] Zhang S., Song X., Zhou J. (2021). An equity and efficiency integrated grid-to-level 2SFCA approach: Spatial accessibility of multilevel healthcare. Int. J. Equity Health.

[4] Idei R., Kato H. (2020). Medical-purposed travel behaviors in rural areas in developing countries: A case study in rural Cambodia. Transportation.

[5] Jin Z., Northridge M.E., Metcalf S.S. (2018). Modeling the influence of social ties and transportation choice on access to oral healthcare for older adults. Appl. Geogr..

[6] Du F., Mao L., Wang J. (2021). Determinants of travel mode choice for seeking healthcare: A comparison between elderly and non-elderly patients. J. Transp. Geogr..

[7] Kwan M. (1999). Gender, the home-work link, and space-time patterns of nonemployment activities. Econ. Geogr..

[8] Medina S., Erath A. (2013). Estimating dynamic workplace capacities by means of public transport smart card data and household travel survey in Singapore. Transp. Res. Rec..

[9] Du F., Wang J., Jin H. (2021). Whether Public Hospital Reform Affects the Hospital Choices of Patients in Urban Areas: New Evidence from Smart Card Data. Int.J. Environ. Res. Public Health.

[10] Wang J., Du F., Huang J., Liu Y. (2020). Access to hospitals: Potential vs. observed. Cities.

[11] Huang J., Levinson D., Wang J., Zhou J., Wang Z. (2018). Tracking job and housing dynamics with smartcard data. Proc. Natl. Acad. Sci. USA.

[12] Deville P., Linard C., Martine S., Gilbert M., Stevens R.F., Gaughan E.A., Blondel D.V., Tatem J.A. (2014). Dynamic population mapping using mobile phone data. Proc. Natl. Acad. Sci. USA.

[13] Chen C., Ma J., Susilo Y., Liu Y., Wang M. (2016). The promises of big data and small data for travel behavior (aka human mobility) analysis. Transp. Res. Part C Emerg. Technol..

[14] Huang J., Levinson D., Wang J., Jin H. (2019). Job-worker spatial dynamics in Beijing: Insights from Smart Card Data. Cities.

[15] Hu Y., Miller H.J., Li X. (2014). Detecting and analyzing mobility hotspots using surface networks. Trans. GIS.

[16] Liu X., Gong L., Gong Y., Liu Y. (2015). Revealing travel patterns and city structure with taxi trip data. J. Transp. Geogr..

[17] Gschwender A., Munizaga M., Simonetti C. (2016). Using smart card and GPS data for policy and planning: The case of Transantiago. Res. Transp. Econ..

[18] Tu W., Cao R., Yue Y., Zhou B., Li Q. (2018). Spatial variations in urban public ridership derived from GPS trajectories and smart card data. J. Transp. Geogr..

[19] Zhang X., Xu Y., Tu W., Ratti C. (2018). Do different datasets tell the same story about urban mobility—A comparative study of public transit and taxi usage. J. Transp. Geogr..

[20] Ghasemlou K., Ergun M., Dadashzadeh N. (2021). Exploring Equity in Public Transportation Planning Using Smart Card Data. Sensors.

[21] Du F., Mao L., Wang J., Jin H. (2020). Inferring transit-based health seeking patterns from smart card data–A case study in Beijing, China. Health Place.

[22] Kong X., Liu Y., Wang Y., Tong D., Zhang J. (2017). Investigating public facility characteristics from a spatial interaction perspective: A case study of Beijing hospitals using taxi data. ISPRS Int. J. Geo-Inf..

[23] Pan X., Kwan M., Yang L., Zhou S., Zuo Z., Wan B. (2018). Evaluating the accessibility of healthcare facilities using an integrated catchment area approach. Int. J. Environ. Res. Public Health.

[24] Commins N., Nolan A. (2011). The determinants of mode of transport to work in the Greater Dublin Area. Transp. Policy.

[25] Brands T., de Romph E., Veitch T., Cook J. (2014). Modelling public transport route choice, with multiple access and egress modes. Transp. Res. Procedia.

[26] Böcker L., Van Amen P., Helbich M. (2017). Elderly travel frequencies and transport mode choices in Greater Rotterdam, the Netherlands. Transportation.

[27] Alsger A., Tavassoli A., Mesbah M., Ferreira L., Hickman M. (2018). Public transport trip purpose inference using smart card fare data. Transp. Res. Part C: Emerg. Technol..

[28] Gong L., Liu X., Wu L., Liu Y. (2016). Inferring trip purposes and uncovering travel patterns from taxi trajectory data. Cartogr. Geogr. Inf. Sci..

[29] Chester M., Fraser A., Matute J., Flower C., Pendyala R. (2015). Parking infrastructure: A constraint on or opportunity for urban redevelopment? A study of Los Angeles County parking supply and growth. J. Am. Plan. Assoc..

[30] Shiftan Y., Barlach Y. (2002). Effect of employment site characteristics on commute mode choice. Transp. Res. Rec..

[31] Kim S., Ulfarsson G.F. (2004). Travel mode choice of the elderly: Effects of personal, household, neighborhood, and trip characteristics. Transp. Res. Rec..

[32] Liu L., Andris C., Ratti C. (2010). Uncovering cabdrivers’ behavior patterns from their digital traces. Comput. Environ. Urban.

[33] Zhang Y., Martens K., Long Y. (2017). Revealing group travel behavior patterns with public transit smart card data. Travel Behav. Soc..

[34] Ma X., Liu C., Wen H., Wang Y., Wu Y. (2017). Understanding commuting patterns using transit smart card data. J. Transp. Geogr..

[35] Santi P., Resta G., Szell M., Sobolevsky S., Strogatz H.S., Ratti C. (2014). Quantifying the benefits of vehicle pooling with shareability networks. Proc. Natl. Acad. Sci. USA.

[36] Wang W., Pan L., Yuan N., Zhang S., Liu D. (2015). A comparative analysis of intra-city human mobility by taxi. Phys. A Stat. Mech. Its Appl..

[37] Kung K.S., Kael G., Stanislav S., Carlo R., Ramasco J.J. (2014). Exploring universal patterns in human home-work commuting from mobile phone data. PLoS ONE.

[38] Louail T., Lenormand M., Ros G.C.O., Picornell M., Herranz R., Frias-Martinez E., Ramasco J.J., Barthelemy M. (2014). From mobile phone data to the spatial structure of cities. Sci. Rep..

[39] Alexander L., Jiang S., Murga M., González M.C. (2015). Origin–destination trips by purpose and time of day inferred from mobile phone data. Transp. Res. Part C Emerg. Technol..

[40] Li M., Dong L., Shen Z., Lang W., Ye X. (2017). Examining the interaction of taxi and subway ridership for sustainable urbanization. Sustainability.

[41] Jia P., Wang F., Xierali I. (2017). Using a Huff-based model to delineate Hospital Service Areas. Professional Geographer.

[42] Du M., Cheng L., Li X., Yang J. (2020). Factors affecting the travel mode choice of the urban elderly in healthcare activity: Comparison between core area and suburban area. Sustain. Cities Soc..

[43] Li X., Zhang Y., Du M. (2018). Analysis of travel decision-making for urban elderly healthcare activities under temporal and spatial constraints. Sustainability.

[44] Yang G., Song C., Shu H., Zhang J., Pei T., Zhou C. (2016). Assessing Patient bypass Behavior Using Taxi Trip Origin-Destination (OD) Data. ISPRS Int. J. Geo-Inf..

[45] Cheng L., Yang M., De Vos J., Witlox F. (2020). Examining geographical accessibility to multi-tier hospital care services for the elderly: A focus on spatial equity. J. Transp. Health.

